# A Study on Curing Kinetics of Nano-Phase Modified Epoxy Resin

**DOI:** 10.1038/s41598-018-21208-0

**Published:** 2018-02-14

**Authors:** Hailing Ma, Xin Zhang, Feifei Ju, Sang-Bing Tsai

**Affiliations:** 1 Zhongshan Institute, University of Electronic Science and Technology of China, Guangdong, 528400 China; 2 School of Mathematics and Physics, Jinggangshan University, Ji’an, 343009 China; 3 State Key Laboratory of Coal Mine Disaster Dynamics and Control, College of Resource and Environmental Sciences, Chongqing University, Chongqing, 400030 China; 4 School of Materials Engineering, Jilin university, Changchun, 130000 China

## Abstract

In this paper, DSC curves at different heating rates were measured by DSC, the characteristic curing temperature was determined, and the optimum curing conditions were obtained. The KAS method, Friedman method and FWO method were used to analyze the DSC curves respectively. The kinetic parameters and the reaction mechanism function of the curing system were obtained, and the results of different analytical methods were compared and analyzed. Result from fitting and verification of the curing kinetic model for the curing system of the nano-phase modified epoxy resin further demonstrates that the nano-particle could play a catalytic role in the curing reaction of the epoxy resin and could reduce the apparent activation energy of the system, thus it is considered as a breakthrough in the field of resin research.

## Introduction

With the advantage of high bonding strength, good dimensional stability and chemical resistance, high mechanical strength, excellent electrical insulation and strong radiation resistance, epoxy resin is widely used as coating, adhesive, electronic electrical material, civil engineering material, engineering plastics, composite material and so on^1,2^. The raw material of bisphenol A epoxy resin is easy to get, with high cost performance. As a kind of glycidyl ether type epoxy resin with the largest output, bisphenol A epoxy resin approximately takes up 90% of total output of epoxy resin in China and accounts for about 80% of total output of epoxy resin in the world. As a result, bisphenol A epoxy resin is known as universal epoxy resin. However, epoxy resin prepolymers which will not be cured under normal temperature and general heating conditions cannot be applied directly. Usually, curing agent or promoter can be introduced and the performance of epoxy resin prepolymers can be improved through the optimization of thermocuring process to turn epoxy resin prepolymers into thermosetting materials with real use value. However, the internal temperature of resin undergoes complicated changes in the curing process, which will usually lead to the phenomena including a large gradient in local temperature and concentration of thermal stress and even damage the internal structure of cured substance^3^. When alkyl etherification reaction is caused by the insufficient dosage of curing agent and the excess of epoxy group, the glass transition temperature (Tg) of cured substance may be too low, which will have an influence on the final use performance. If curing agent is excessive, residual curing agent may have an impact on curing system^4^. Therefore, it is very important to look for the appropriate equivalence ratio of epoxy resin/curing agent and design reasonable thermocuring programs. Amine curing agent is the most important category of epoxy resin curing agents, whose performance determines the use performance of epoxy resin to a large extent. Aliphatic amine curing agent is a type of widely-used curing agents. However, these cured substances are far less than aromatic curing agents due to their generally poor heat resistance^5^.

Epoxy resin is an epoxy oligomer, which can form a three-dimensional network of thermosetting material when it reacts with the curing agent^6^. Epoxy resin crosslinking curing process is generally divided into three types: free radical polymerization, condensation reaction and addition reaction types. The common feature is that it releases the chemical reaction heat during the curing reaction. Epoxy resin undergoes complex physical and chemical reactions and complex chemical reaction kinetics processes during crosslinking and curing^7–9^. It is very important to study the mechanism of curing process, in order to reveal the mechanism of curing reaction, and control the curing reaction process. It is also important to optimize the curing process parameters. There are many ways to study the curing process, among which the thermal analysis method is the major one.

Thermal analysis method is used to study the solid process through the thermal tracking detection of polymer materials in chemical reaction^10–14^. Thermal analysis kinetics is to use the thermal analysis technology to study the physical changes and chemical reactions, by means of a certain mathematical approach to obtain the corresponding reaction of the kinetic parameters and reaction mechanism^15–21^. There are two methods of thermal analysis kinetics: isothermal and non-isothermal methods. The non-isothermal method has many advantages over the conventional isothermal method^22^. So it gradually becomes the main method of thermal analysis kinetics. Non-isothermal method is divided into the single scanning rate non-isothermal method and the multiple scanning rate non-isothermal method^23,24^.

Multiple scanning rate non-isothermal method refers to using different heating rates measured under a number of dynamic curves to carry out the method of dynamic analysis^25,26^. According to the different mathematical methods, it can be divided into two categories of differential and integral methods. At present, in the method of research dynamics, the differential method is mainly represented by Kissinger-Akahira-Sunose (KAS) method and Friedman method, and the integral method is mainly represented by Flynn-Wall-Ozawa (FWO) method^20,23,27–30^.

The KAS method is based on the assumption that the maximum rate of the curing reaction occurs at the peak temperature of the curing reaction exothermic peak, so the KAS method is also called as the maximum rate method^31^. Friedman method and FWO method are used in a same conversion of several kinetic curves of the data. They are is also called as the Isoconversional method, which can obtain more reliable values of the activation energy E in the premise of not involving the kinetic model function eliminating the effects of model function in calculating Arrhenins parameters. So it is also known as a model free method. As the results show that the multiple scanning rate method and the equal conversion method can describe the complex solid - state reaction kinetics more effectively.

The epoxy resin kinetic model parameters are typically obtained by differential scanning calorimetry (DSC)^32^. The advantages of this method include use of less sample, convenient operation and strong ability of data analysis, particularly suitable for thermosetting resin curing reaction kinetics of the study.

The 21st century features robust development of macromolecular materials, among which the epoxy resin is the pilot in the development and application in this area. Epoxy resin is widely used in electrics, semiconductor electronics, special coating aeronautics, astronautics, etc., owing to its excellent properties. The curing kinetics analysis on epoxy resin is helpful to better understand the characteristics of its system, the curing conditions and the impact imposed on the curing activities by the dimensions of curing molds. It can also help to establish a quantitative relation between the curing conditions, epoxy resin, as well as the chemical structure of curing agents and the properties of curing objects to reduce the experiments in order to optimize the curing process.

As an amorphous viscous liquid, epoxy resin is plastic when heated, without clear melting point, becoming soft when heated, turning sticky when gradually melted, insoluble in water, and will not harden by itself. Therefore, it almost has no value to use independently. It must first react with curing agents to create a polymer with a three-dimensional structure which does not melt or dissolve, and then it can be usable. When a certain amount of curing agents is added, it gradually hardens and turns into chemical materials with distinct properties. Therefore, curing agents must be added to react under certain conditions to turn into a cross-linked network structure in order to create formula resin. In this way, the epoxy material can display excellent properties and be truly usable. The common amine curing agents are: ethylenediamine, diethylene polyamine, and polyethylene polyamine, etc.

## Experimental Study

### Curing Principle

Ammonium hexafluoroantimonate is a kind of Cationic curing agent by heated, which could trigger low temperature and curing speed, and dissolve in epoxy resin. Curing products have better adhesion and corrosion resistance, anti-aging properties.

**Figure 1:**
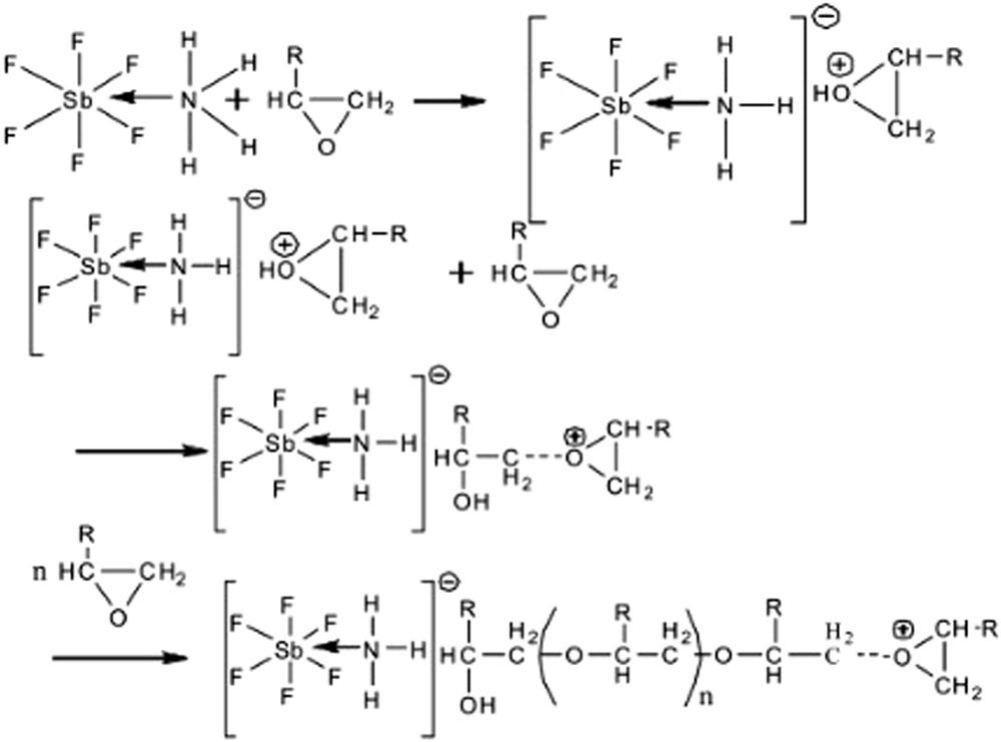
Curing principle.

The curing mechanism is shown in Fig. 1.

Firstly, epoxygroups in the epoxy group react with ammonium hexafluoroantimonate to capture its active hydrogen, to generate oxygenium ions, with hydroxyl groups of oxygen ions as the initiator, and epoxy groups continue to react, to open the way for chain ring polymerization of curing epoxy resin. As long as the epoxy repair agent is in contact with the curing agent, the curing reaction can be automatically carried out. Because the curing agent can be well dissolved in the epoxy resin, it could ensure that micro-cracks derive from self-healing, and achieve a high polymerization rate and polymerization degree.

### Curing Process and Conclusion

**Figure 2:**
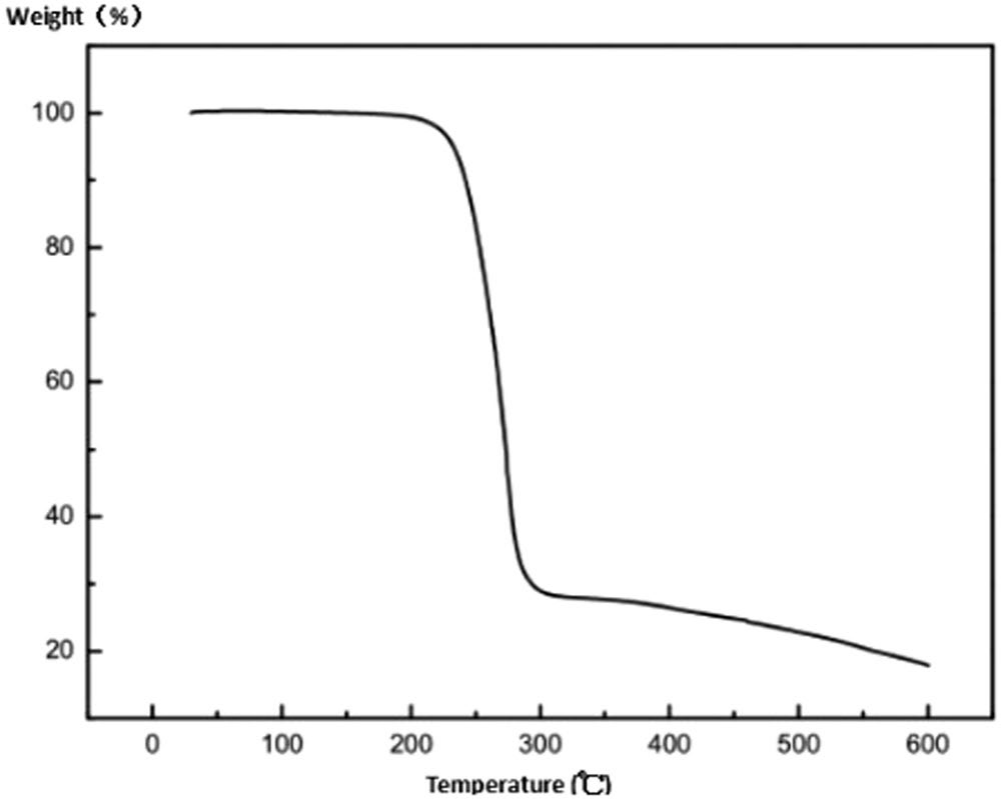
Thermal weight loss curve of curing agent.

Figure 2 shows the thermogravimetric curve of the curing agent. It can be seen that the weight loss starting temperature is 254.4 °C, which indicates that the ammonium hexafluoroantimonate curing agent has good thermal stability and meets its requirement of stability in the self-repairing system. Curing agent in the matrix can exist with stability for a long term.

**Figure 3:**
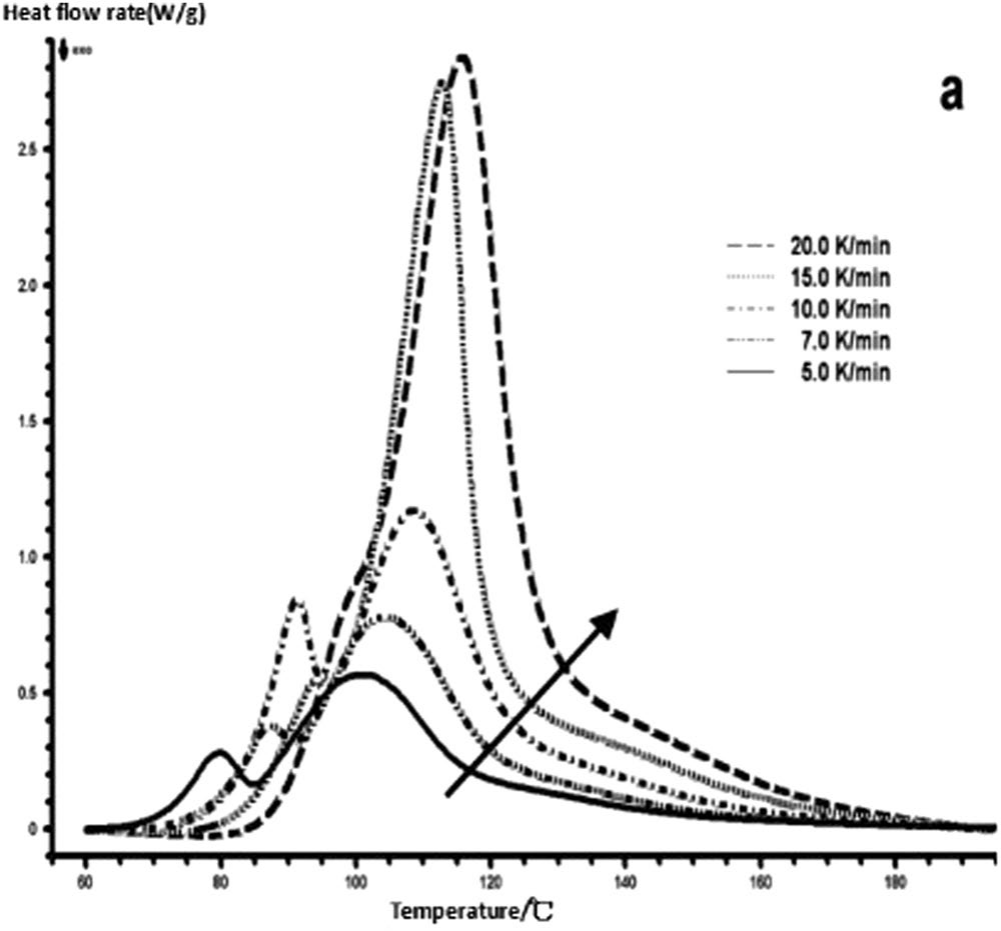
3 Dynamic curing DSC curves for different curing systems-a: pure epoxy exothermic peak with temperature curve.

**Figure 4:**
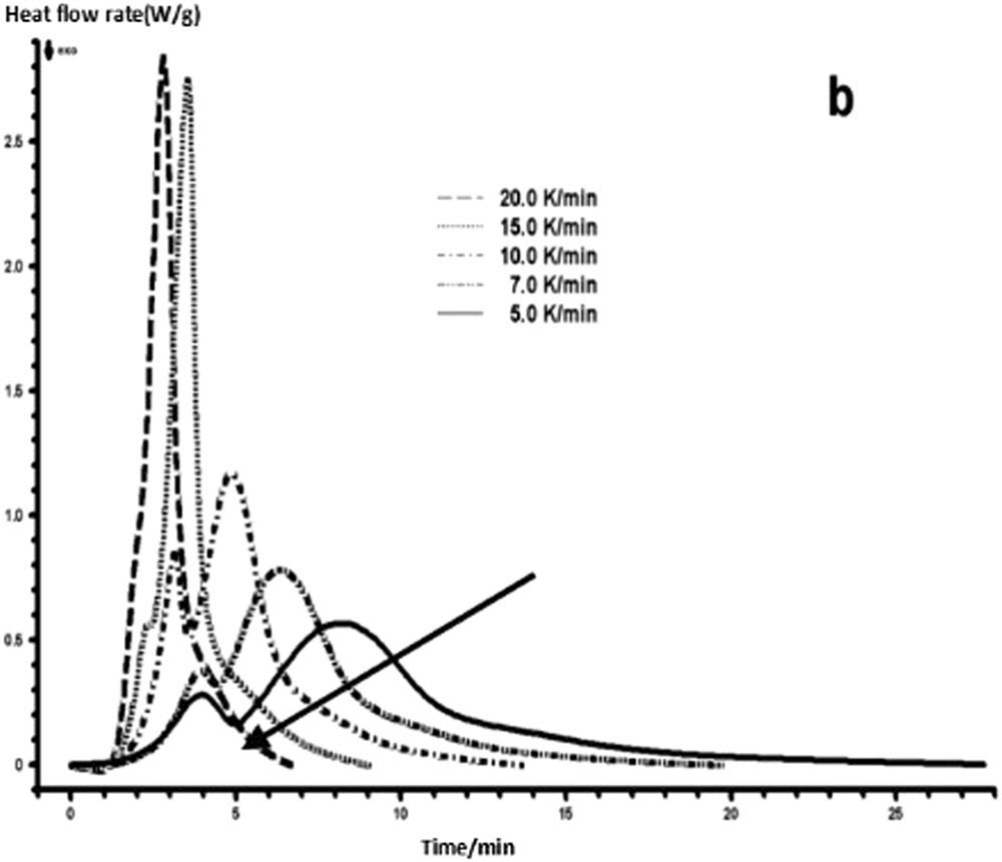
3 Dynamic curing DSC curves for different curing systems-b: pure epoxy exothermic peak with time curve.

**Figure 5:**
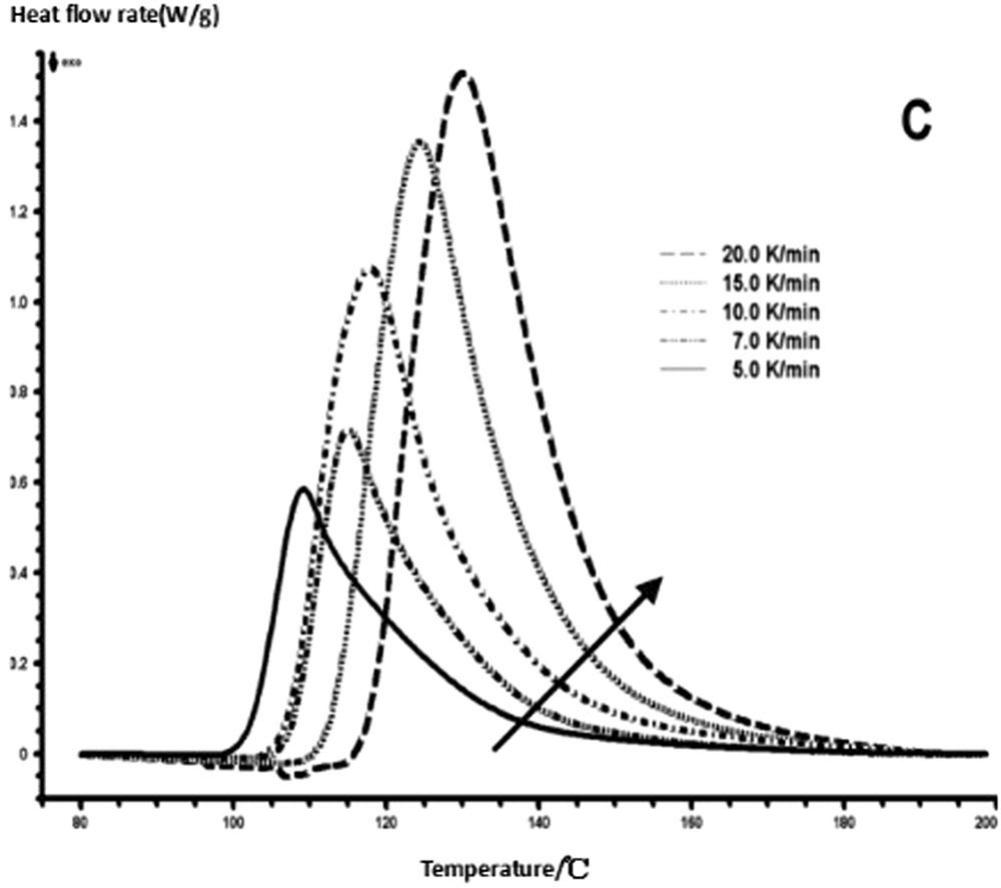
3 Dynamic curing DSC curves for different curing systems-c: modified epoxy resin exothermic peak with temperature curve.

**Figure 6:**
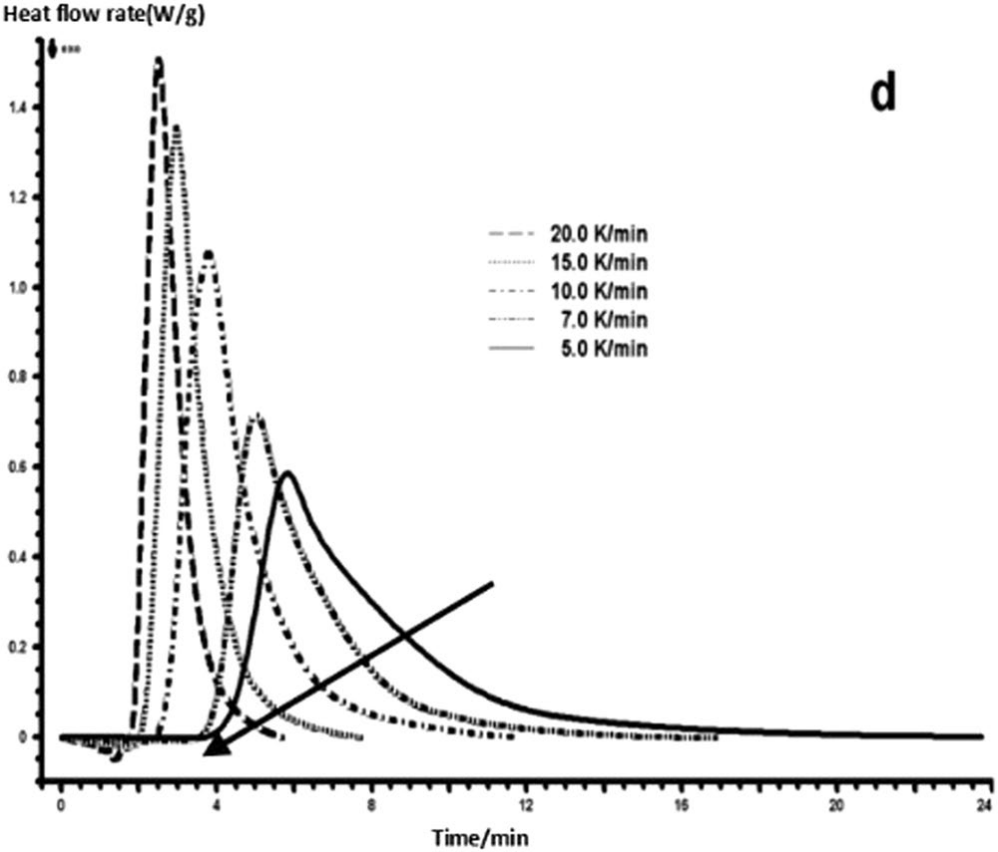
3 Dynamic curing DSC curves for different curing systems-d: modified epoxy exothermic peak with time curve.

The curing reaction exothermic peak of the epoxy resin and the nano rubber modified epoxy resin system are related to the curing temperature and time in different heating rates, as shown in Figs 3–6, in which the arrow direction is the increasing direction of the heating rate. It can be seen from Figs 3–6 that the curing reaction characteristic temperature of each curing system is closely related to the heating rate. From the curve of the exothermic peak with time, it can be seen that with the increase of the heating rate, the exothermic peak becomes steeper gradually, and the curing time is obviously shortened. From the change curve of the exothermic peak to the temperature (Figs 3a and 5c), it can be seen that the curing initiation temperature and the peak temperature of the system move toward the high temperature with the increase of the heating rate.

This shows that the curing reaction is not only a thermodynamic process, but also a dynamic process. At a lower heating rate, the reaction system has sufficient time to react, so the reaction starts at the lower temperature. With the increase of the reaction rate, the ratio *dH/dt* of the system becomes larger, which means the increase of the thermal effect per unit time and the thermal inertia. The greater the temperature difference is, the higher the temperature of the curing reaction exothermic is. After the addition of nano-particles, the exothermic peak can be seen to be sharpened (Figs 5c and 6d), and the addition of the nano-particles to the pure epoxy resin increases from the original bimodal to the single peak. Based on the peak value of heat peak, it can be seen that the amount of heat released during the reaction is obviously reduced and the reaction time is shorter. It shows that the curing reaction becomes gentler and the reaction rate is accelerated. The nano-particles can promote the curing of epoxy resin.

### Analysis of Curing System of Curing System

Kissinger-Akahira-Sunose (KAS) equation is as follows:

1 $$\mathrm{ln}(\frac{{\rm{\beta }}}{{{\rm{T}}}_{{\rm{p}}}^{2}})=\,\mathrm{ln}\,\frac{{\rm{AR}}}{{\rm{E}}}-\frac{{\rm{E}}}{{\rm{R}}}\frac{1}{{{\rm{T}}}_{{\rm{p}}}}$$

**Figure 7:**
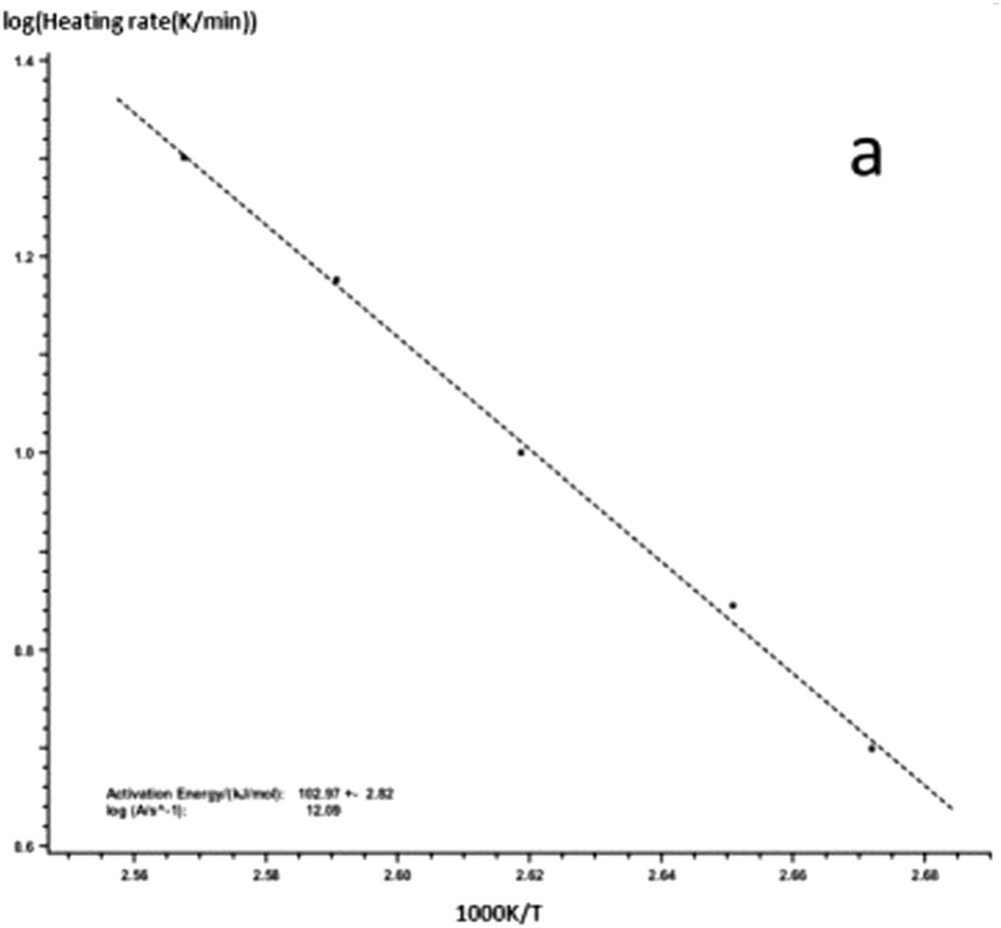
KAS method to calculate the linear regression curve of curing reaction -a: 618 epoxy resin.

**Figure 8:**
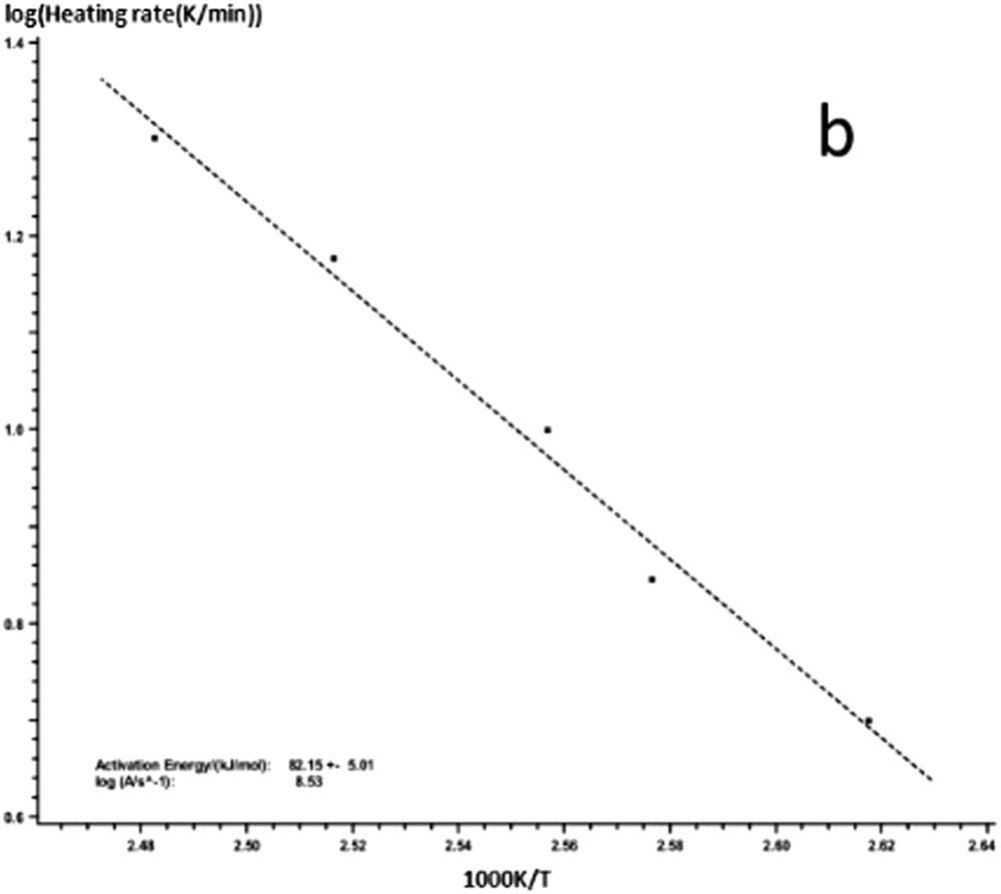
KAS method to calculate the linear regression curve of curing reaction -b: nano-phase modified epoxy resin.

In the formula, β is the heating rate, T_P_ is the curing peak temperature, E is the apparent activation energy, A is the pre-index factor, R is the universal gas constant. We can use $(\frac{{\rm{\beta }}}{{{\rm{T}}}_{{\rm{p}}}^{2}})$ to map $\frac{1}{{{\rm{T}}}_{{\rm{p}}}}$, you can get a straight line from the slope of the straight line, and obtain the intercept from the apparent activation energy E and pre-exponential factors A. According to the DSC curve of the graph and the peak temperature T_p_ at different heating rates for each curing system the linear regression of $\frac{1}{{{\rm{T}}}_{{\rm{p}}}}$ with $(\frac{{\rm{\beta }}}{{{\rm{T}}}_{{\rm{p}}}})$ is given in Figs 7 and 8.

The *E* and *A* of each curing system are obtained from the slope and intercept of the straight line in the figure. The results are shown in Table 1. The different curing systems *E* and *A* can be seen by KAS method. The results show that the E and A of the curing system are obviously decreased after the addition of the nanoparticles, which indicates that the reaction system is more rapid after adding the nanoparticles.

**Table 1:** The activation energy and the pre - exponential factor of the curing system.

Curing system	E	logA
Pure epoxy resin	102.97 + −2.82	12.09
Nano - phase modified epoxy resin	82.15 + −5.01	8.53

### Analysis of Curing System by Friedman Method

Friedman equation is as follows:

2 $$\mathrm{ln}(\frac{\beta {\rm{d}}\alpha }{{\rm{dT}}})=\,\mathrm{ln}\,{\rm{Af}}({\rm{\alpha }})-\frac{{\rm{E}}}{{\rm{RT}}}$$

In the formula, the physical meaning of β, E, A, R, α is the conversion rate, and f(α) is the differential form of the reaction mechanism function. Friedman equation E and A can be obtained by the following method. The specific steps are as follows: Since the same conversion rate α is selected at different heating rates β, tthe integral form of reaction mechanism function in thehen f(α) is a constant value, so that $\mathrm{ln}(\frac{\beta {\rm{d}}\alpha }{{\rm{dT}}})$ and $\frac{1}{{\rm{T}}}$ are linearly related, and the slope and intercept of the line are given with the corresponding E and A values of α, for each α can be a set of E and A values, all of which could be sued to make a logical analysis, you can determine the E and A value.

**Figure 9:**
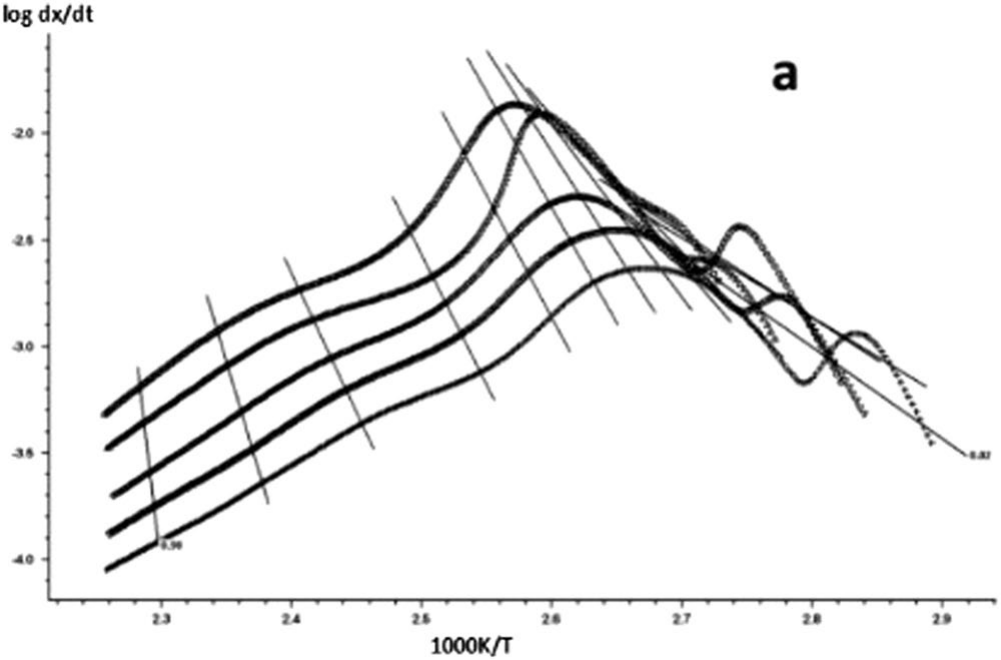
The linear regression curve of the curing reaction calculated by the Friedman method-a: 618 epoxy resin.

**Figure 10:**
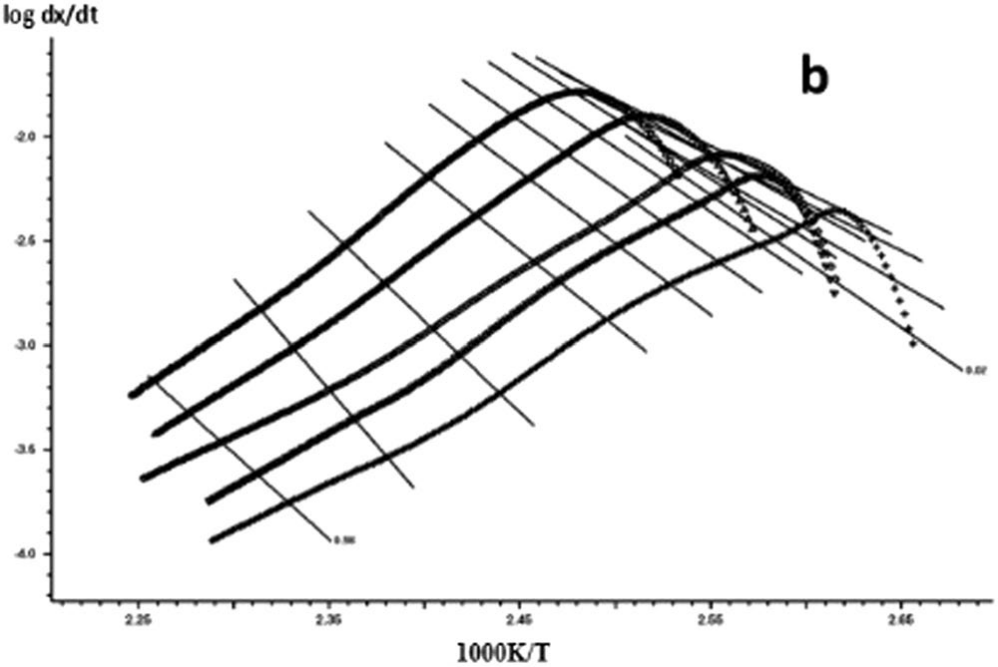
The linear regression curve of the curing reaction calculated by the Friedman method-b: nano-phase modified epoxy resin.

**Figure 11:**
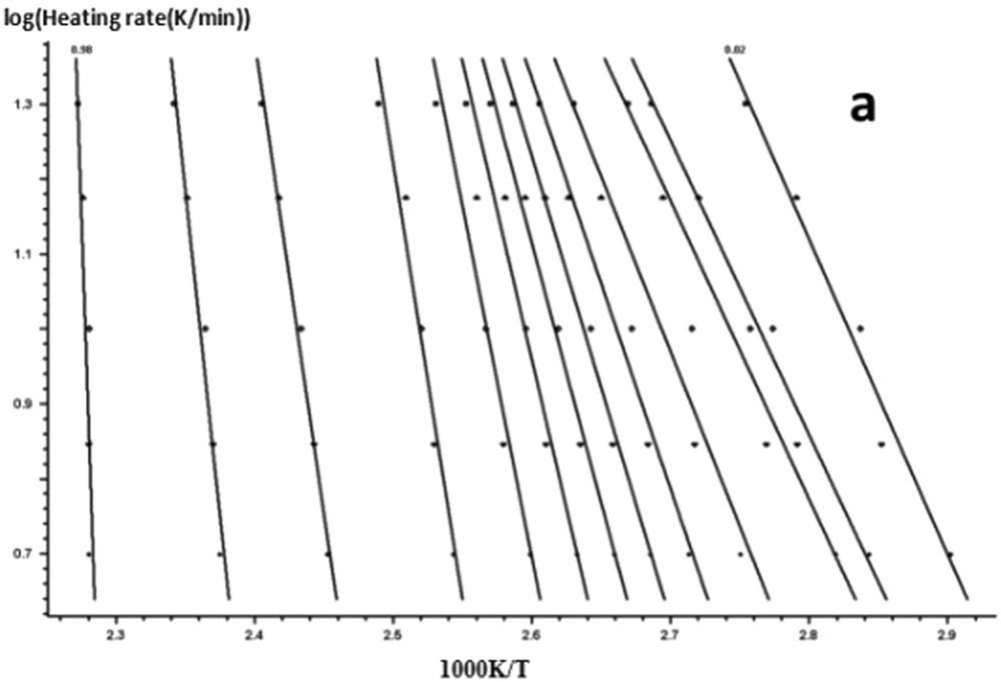
Friedman method analysis results -a: 618 epoxy resin.

**Figure 12:**
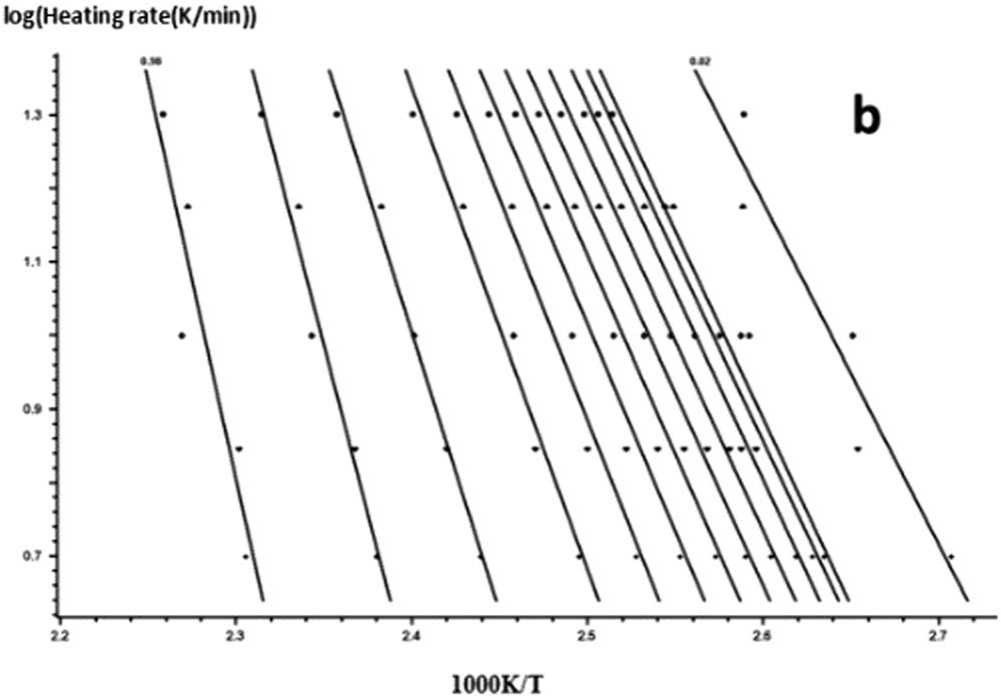
Friedman method analysis results -b: nano-phase modified epoxy resin.

The DSC curve in Figs 3 and 6 was analyzed according to the Friedman equation. We can get the regression curve by $\mathrm{ln}(\frac{\beta {\rm{d}}\alpha }{{\rm{dT}}})$ and $\frac{1}{{\rm{T}}}$ mapping and computer fitting as shown Figs 9 and 10. For each curing system, the slope and intercept of the linear regression line at each conversion rate were used to obtain the E and A values at the corresponding conversion rate. The results are shown in Figs 11 and 12, and the specific data are shown in Table 2.

**Table 2:** Friedman method analysis results.

Conversion rate	618 epoxy resin		Nano - phase modified epoxy resin	
	E	lgA	E	lgA
0.02	82.11 + −8.89	9.01	121.89 + −7.52	13.97
0.05	71.22 + −9.07	7.58	101.60 + −7.50	11.38
0.10	76.53 + −22.18	8.37	87.67 + −6.21	9.64
0.2	122.04 + −4.72	14.75	85.33 + −4.95	9.42
0.3	135.22 + −8.68	16.60	97.81 + −9.49	11.09
0.4	155.86 + −13.87	19.43	110.81 + −13.77	12.78
0.5	182.12 + −25.23	22.95	118.53 + −13.79	13.72
0.6	207.40 + −40.57	26.21	124.69 + −11.32	14.43
0.7	220.30 + −32.00	27.57	131.22 + −8.18	15.15
0.8	234.78 + −13.80	28.78	134.86 + −6.25	15.44
0.9	249 + −17.66	29.65	155.12 + −12.15	17.62
0.95	392.83 + −44.42	46.45	179.15 + −31.19	20.17
0.98	1009.49 + −349.50	118.91	127.83 + −48.02	13.61

It can be seen from Figs 11 and 12 and Table 2 that the activation energy at the beginning of the reaction is slightly higher for the pure epoxy resin, and the apparent activation energy at the low conversion rate stage is greatly reduced because the curing agent needs a process of dissociation that could produce a curing effect, during which to the energy needs to be dissipated. When the conversion rate is more than 20%, the E and A of the system increase with the increase of the conversion rate, and the apparent activation energy deviation also gradually rises. When the conversion rate is more than 90%, the apparent activation energy can be steeply increased, indicating that the epoxy resin has a gel effect, the viscosity of the system increases sharply, and the reaction functional groups close to the gel point are bound in the crosslinking network, the energy required to react with each other between the reaction functional groups increases, so that the latter stage of the curing reaction requires a great amount of energy until the end of the curing. The apparent activation energy of epoxy resin after nano-phase modification is stable, and it is lower than that of pure epoxy resin in different conversion stages. It shows that the curing reaction is gentle, and the presence of nanoparticles for the curing reaction plays a catalytic role. The change trend of lgA is similar to apparent activation energy E.

The E and A data obtained by Friedman analysis in Table 2 are averaged to obtain the apparent activation energy E and the pre-exponential factor A for each solidified system. The results are shown in Table 3. It can be seen that the addition of organic nanoparticles, the apparent activation energy of the system and the pre-exponential factors have a significant reduction, indicating that the nano-particles on the curing play a catalytic role.

**Table 3:** The apparent activation energy and pre-exponential factors of the curing system.

Curing system	E	lgA
Pure epoxy resin	157.94	19.17
Nano - phase modified epoxy resin	115.41	13.15

### Analysis of Curing System by FWO Method

Flynn-Wall-Ozawa (FWO) equation is as follows:

3 $$\mathrm{lg}\,{\rm{\beta }}=\,{\rm{lg}}(\frac{{\rm{AE}}}{{\rm{RG}}({\rm{\alpha }})})-2.315-0.4567\frac{{\rm{E}}}{{\rm{RT}}}$$

As we know, G(α) is the integral form of reaction mechanism function in the formula. There are formulas that uses FWO method to find E, A. Due to the same conversion rate α is chosen at different heating rates β, G(α) is a constant value in the formula, and in this situation, lgβ and $\frac{1}{{\rm{T}}}$ have a linear relationship. As the Friedman method is adopted, the corresponding slope and intercept can be used to find the E and A values corresponding A. For each α can be a set of E and A values, for all numerical averages, you can determine the reasonable E and A value.

**Figure 13:**
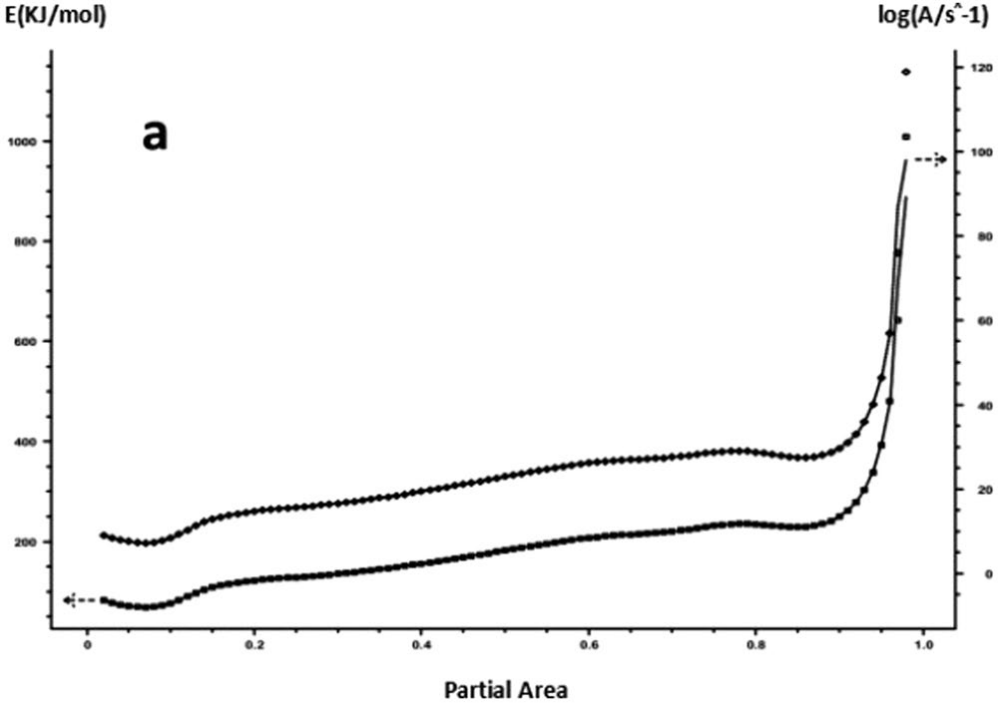
FWO method for calculating the linear regression curve of the curing reaction -a: 618 epoxy resin.

**Figure 14:**
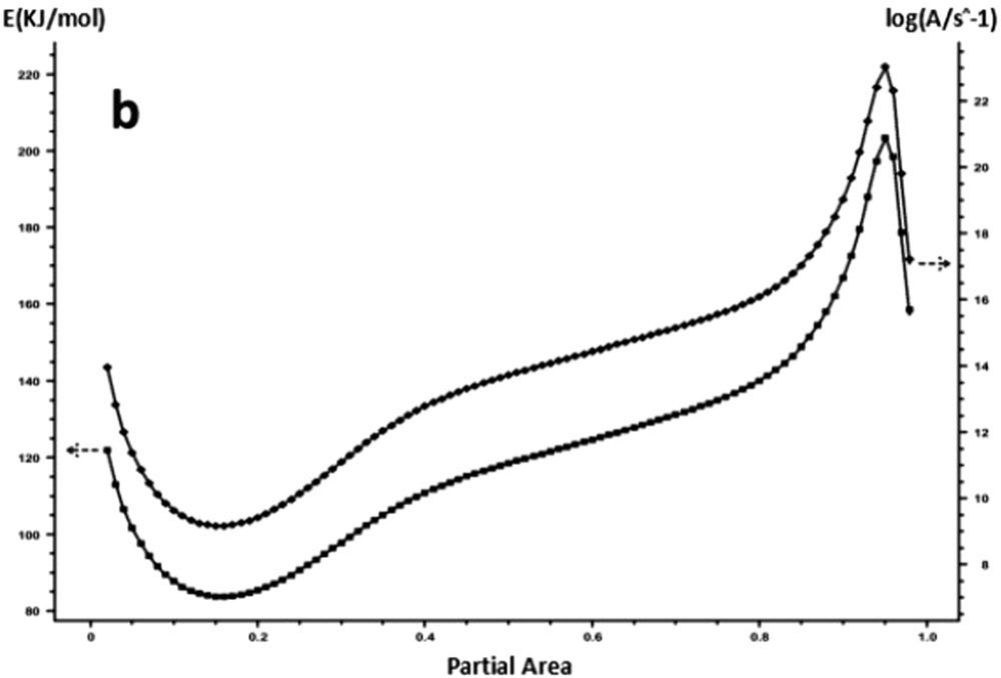
FWO method for calculating the linear regression curve of the curing reaction -b: nano-phase modified epoxy resin.

According to the FWO equation, the DSC curve of the graph is analyzed, and the linear regression curves of Figs 13 and 14 are obtained by linear fitting with lgβ and $\frac{1}{{\rm{T}}}$, linear regression and computer.

**Figure 15:**
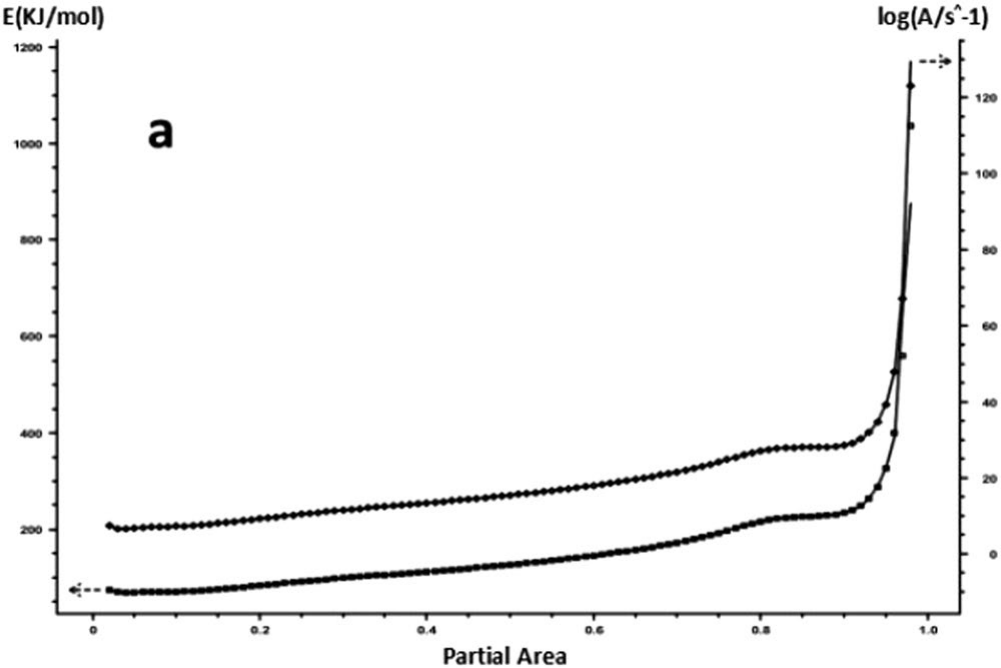
FWO method analysis results -a: 618 epoxy resin.

**Figure 16:**
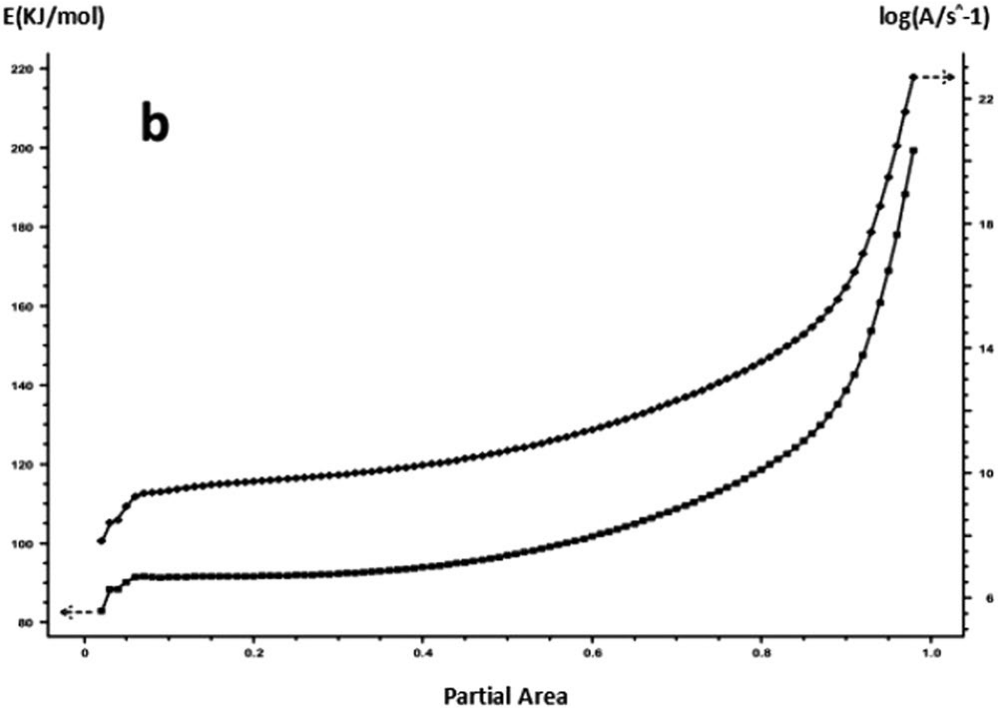
FWO method analysis results -b: nano-phase modified epoxy resin.

For each curing system, using the slope and intercept of the regression curve at each conversion rate, the corresponding conversion rates E and A were obtained. The results are shown in Figs 15 and 16 and Table 4.

**Table 4:** FWO analysis results.

Conversion rate	618 epoxy resin		Nano - phase modified epoxy resin	
	E	lgA	E	lgA
0.02	75.07 + −6.42	7.51	82.86 + −14.17	7.82
0.05	69.61 + −5.29	6.82	90.24 + −13.44	8.94
0.10	70.74 + −6.86	7.22	91.41 + −11.61	9.45
0.2	83.49 + −12.28	9.21	91.72 + −10.74	9.73
0.3	99.25 + −8.33	11.52	92.32 + −10.32	9.95
0.4	112.24 + −6.01	13.39	93.89 + −10.38	10.25
0.5	126.81 + −7.54	15.43	96.90 + −10.93	10.71
0.6	145.64 + −13.90	18.01	101.73 + −11.67	11.39
0.7	172.29 + −26.34	21.56	108.71 + −11.76	12.32
0.8	216.18 + −22.12	27.10	118.60 + −10.30	13.57
0.9	234.25 + −15.06	28.56	138.53 + −8.19	15.96
0.95	326.74 + −36.96	39.34	168.87 + −16.66	19.48
0.98	1037.32 + −367.06	123.06	199.38 + −46.55	22.69

As it can be seen from Table 4, for each curing system, with the increase in conversion rate, the system E and A as a whole shows an increasing trend, and the apparent activation energy deviation gradually increases. Trends are consistent with Friedman’s analysis.

The Eand A values obtained by the FWO method in Table 4 were averaged to obtain the apparent activation energ E and the pre-exponential factor A of the solidified system as a whole. The results are shown in Table 5.

**Table 5:** The apparent activation energy and pre-exponential factors of the curing system.

Curing system	E	lgA
Pure epoxy resin	127.78	15.12
Nano - phase modified epoxy resin	92.32	10.92

Therefore, FWO method is used to analyze the system of E. The addition of organic nano particles, the apparent activation energy and pre-exponential factor decreases significantly, which indicated that nanoparticles to the same catalytic have an effect on curing up.

### Determination of curing reaction series

The reaction series of the curing reaction can be obtained by the Crane equation.

4 $$\frac{{\rm{d}}(\mathrm{ln}\,{\rm{\beta }})}{{\rm{d}}(1/{{\rm{T}}}_{{\rm{p}}})}=-\frac{{\rm{E}}}{{\rm{nR}}}$$

Which the physical meaning of β, T_p_, E, R is the same as above, the reaction series is n. The above formula can be integrated,

5 $$\mathrm{ln}\,{\rm{\beta }}=-\frac{{\rm{E}}}{{\rm{nR}}}\times \frac{1}{{{\rm{T}}}_{{\rm{p}}}}+{\rm{C}}$$

**Figure 17:**
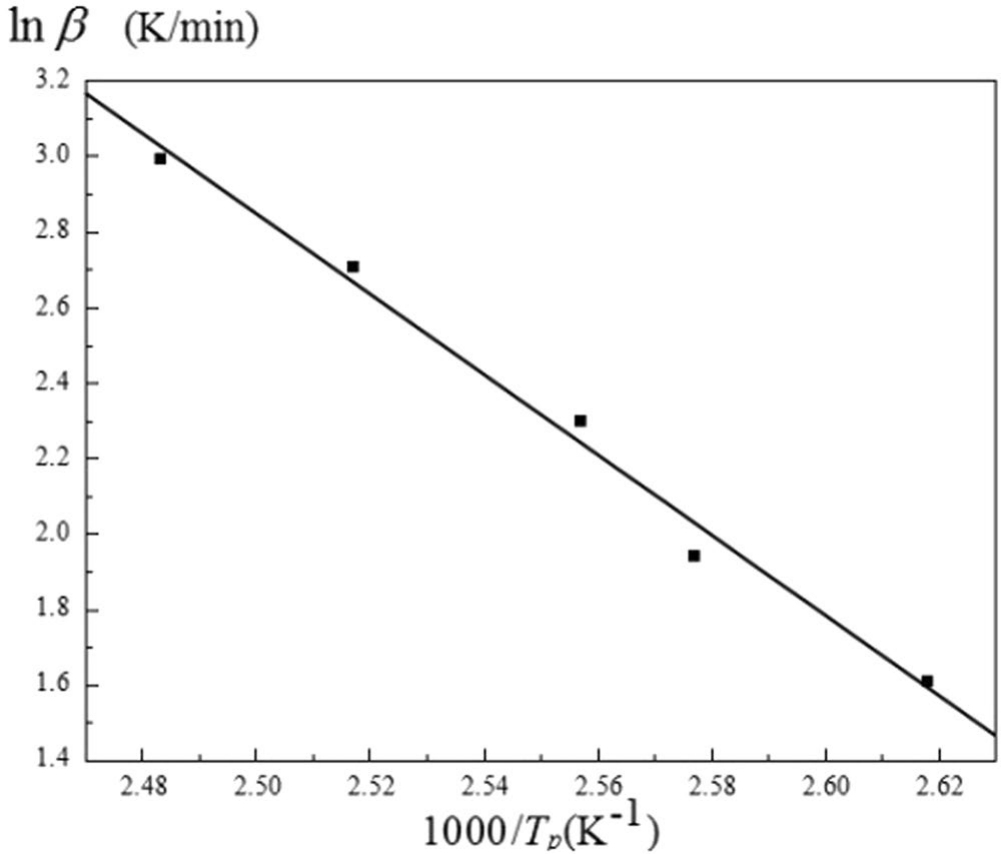
Curing system lnβ-T_p_ linear regression curve.

According to the formula, lnβ to $\frac{1}{{{\rm{T}}}_{{\rm{p}}}}$ linear fit, the results shown in Fig. 9. As can beseen from Fig. 17, the curing system lnβ has a good linear relationship with $\frac{1}{{{\rm{T}}}_{{\rm{p}}}}$, according to the Crane equation, the apparent activation energy of the cured system was analyzed by KAS method, Friedman method and FWO method, and the reaction order of curing system was obtained by different methods, as shown in Table 6.

**Table 6:** three methods to analyze the reaction series.

	KAS	Friedman	FWO
Reaction series	0.93	1.31	1.05

It can be seen that the apparent activation energy obtained by the KAS method, the Friedman method and the FWO method are not all integers, indicating that the curing reaction is a complex reaction and the results of the three methods are also correct.

### Model validation

The commonly used kinetic equation for nonhomogeneous systems under non-isothermal conditions is shown as follows:

6 $$\frac{{\rm{d}}{\rm{\alpha }}}{{\rm{dT}}}=\frac{{\rm{A}}}{{\rm{\beta }}}\exp (-\frac{{\rm{E}}}{{\rm{RT}}}){\rm{f}}({\rm{\alpha }})$$

In this formula, $\frac{{\rm{d}}{\rm{\alpha }}}{{\rm{dT}}}$ for the reaction rate, A for the pre-exponential factor, E for the apparent activation energy, f(α) for the reaction model coefficient, R for the ideal gas constant. ${\rm{\beta }}=\mathrm{dt}/\mathrm{dT}$ for the heating rate.

The above equation can be transformed into a linear equation:

7 $$\mathrm{ln}\,\frac{{\rm{d}}\alpha /{\rm{dT}}}{{\rm{f}}({\rm{\alpha }})}=\,\mathrm{ln}(\frac{{\rm{A}}}{{\rm{\beta }}})-\frac{{\rm{E}}}{{\rm{RT}}}$$

The equation can be seen as ${\rm{y}}={{\rm{a}}}_{0}+{{\rm{a}}}_{1}{\rm{x}}$, in which ${\rm{x}}=1/{\rm{T}}$ can be analyzed using multiple linear programming. The results are shown in Table 7.

**Table 7:** Multiple linear regression analysis results.

Reaction model	Model designation	*f*(*α*)	Variables
Fist-order	F1	(1 − α)	A, E
Second-order	F2	(1 − α)^2^	A, E
nth-order	Fn	(1 − α)^2^	A, E, n
nth-order with autocatalysis	Cn	(1 − α)^n^(1 + K_cat_α)	A, E, n, K_cat_
Prout-Tompkins equation(autocatalytic)	PT	(1 − α)^n^α^m^	A, E, n, m

Based on the function expression of different reaction models f(α), the computer being used to analyze the data fits with the commonly used dynamic mechanism function to obtain the most suitable curing kinetic model.

Five different reaction models in the table were used to fit the previous data using the multivariate least squares regression method. The correlation coefficient (r) is defined as follows:

8 $${\rm{LSQ}}={\sum }_{{\rm{j}}=1}^{{\rm{S}}}{\sum }_{{\rm{k}}=1}^{{{\rm{N}}}_{{\rm{S}}}}{({{\rm{Y}}}_{{\rm{jk}}}-{{\rm{y}}}_{{\rm{jk}}})}^{2}$$

9 $${\rm{r}}=\sqrt{1-\frac{{\rm{LSQ}}}{{\sum }_{{\rm{s}}}({\sum }_{{\rm{k}}}{{\rm{Y}}}_{{\rm{sk}}}^{2}-{({\sum }_{{\rm{k}}}{{\rm{Y}}}_{{\rm{sk}}})}^{2}/{{\rm{N}}}_{{\rm{s}}}}}$$

In the formula: Y_jk_ is the measured value, Y_jk_ is the regression value, S is the number of measurements under different conditions, N_S_ is the number of data measured at one time.

10 $${{\rm{F}}}_{\exp }({{\rm{f}}}_{1},{{\rm{f}}}_{2})=\frac{{\sum }_{{\rm{j}}=1}^{{\rm{S}}}{\sum }_{{\rm{k}}=1}^{{{\rm{N}}}_{{\rm{S}}}}{({{\rm{Y}}}_{{\rm{jk}}}-{{\rm{y}}}_{{\rm{jk}}}({{\rm{model}}}_{1}))}^{2}/{{\rm{f}}}_{1}}{{\sum }_{{\rm{j}}=1}^{{\rm{S}}}{\sum }_{{\rm{k}}=1}^{{{\rm{N}}}_{{\rm{S}}}}{({{\rm{Y}}}_{{\rm{jk}}}-{{\rm{y}}}_{{\rm{jk}}}({{\rm{model}}}_{2}))}^{2}/{{\rm{f}}}_{2}}$$

Which f1 is the degree of freedom of model 1, f_2_ is the degree of freedom of model 2 (reference model). The F_exp_ value is compared with ${{\rm{F}}}_{{\rm{crit}}}({{\rm{f}}}_{1},{{\rm{f}}}_{2})$ at a certain confidence level. The relational expression ${{\rm{F}}}_{\exp } < {{\rm{F}}}_{{\rm{crit}}}({{\rm{f}}}_{1},{{\rm{f}}}_{2})$ indicates no data differences.

The experimental data were fitted and the results are shown in Table 8, the results are shown in Table 9.

**Table 8:** Multiple linear regression analysis results.

Model designation	ln[A] (S^−1^)	E (KJ/mol)	n	lgK_cat_	m	Correlation coefficient
F1	16.8585	141.9868	—	—	—	0.8963198
F2	25.7794	208.3932	—	—	—	0.9151101
Fn	26.6926	215.2044	2.1102	—	—	0.9151968
Cn	6.9441	80.2586	2.5744	2.6125	—	0.982009
PT	9.6277	81.7927	2.334	—	0.8238	0.985467

**Table 9:** F-test statistical analysis results.

Model desgination Statistical analysis	${{\rm{F}}}_{\exp }^{{\rm{\alpha }}}$				
	PT 1.00	Cn 1.24	Fn 5.64	F2 5.64	F1 6.83

${}^{{\rm{a}}}{\rm{F}}_{{\rm{crit}}}=1.08$ for a 95% confidence level.

Comparing the results of 5 commonly used dynamic mechanism function fitting, we can see that the curing reaction of epoxy resin modified by nano phase belongs to the PT kinetic mechanism function.

**Figure 18:**
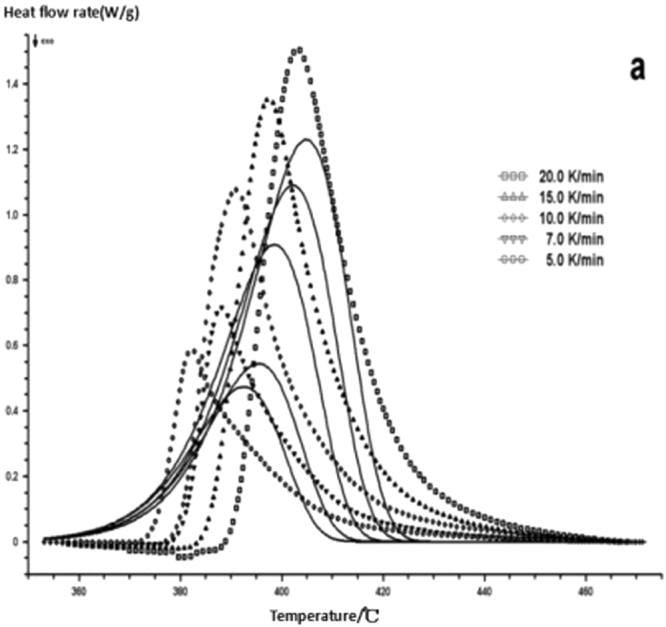
Reaction mechanism function fitting curve-a.

**Figure 19:**
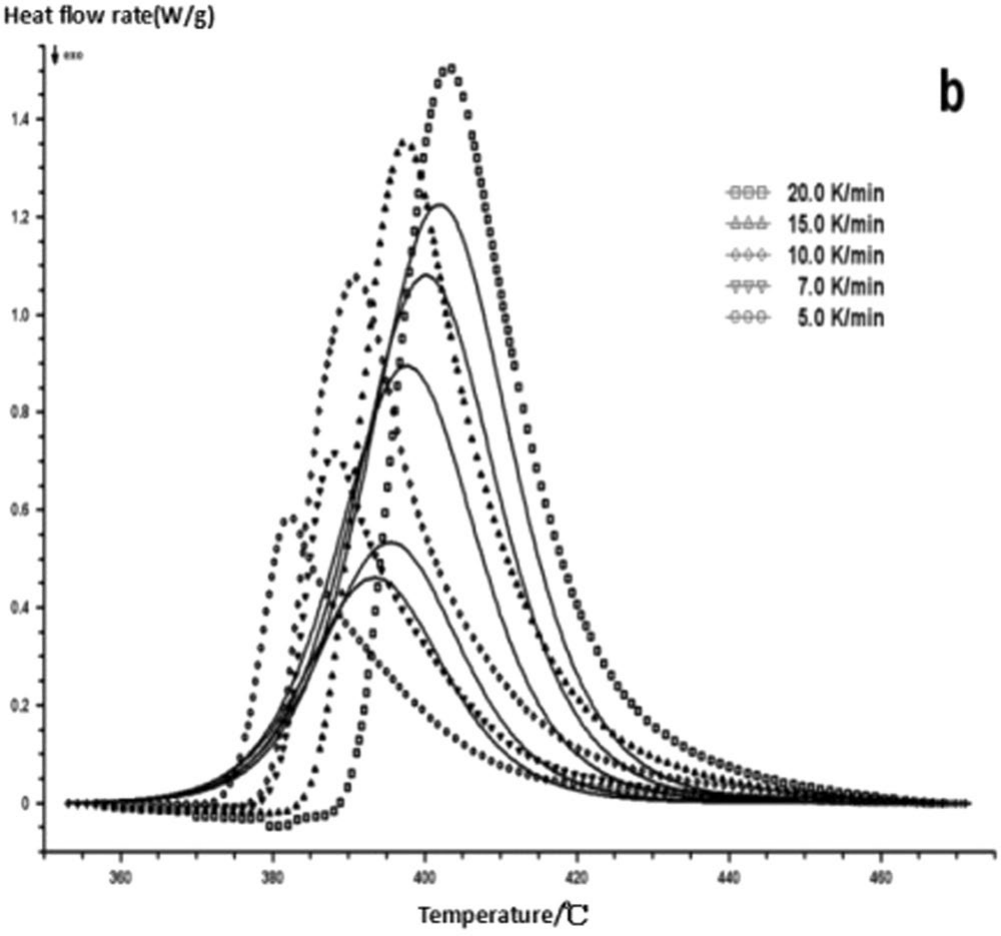
Reaction mechanism function fitting curve-b.

**Figure 20:**
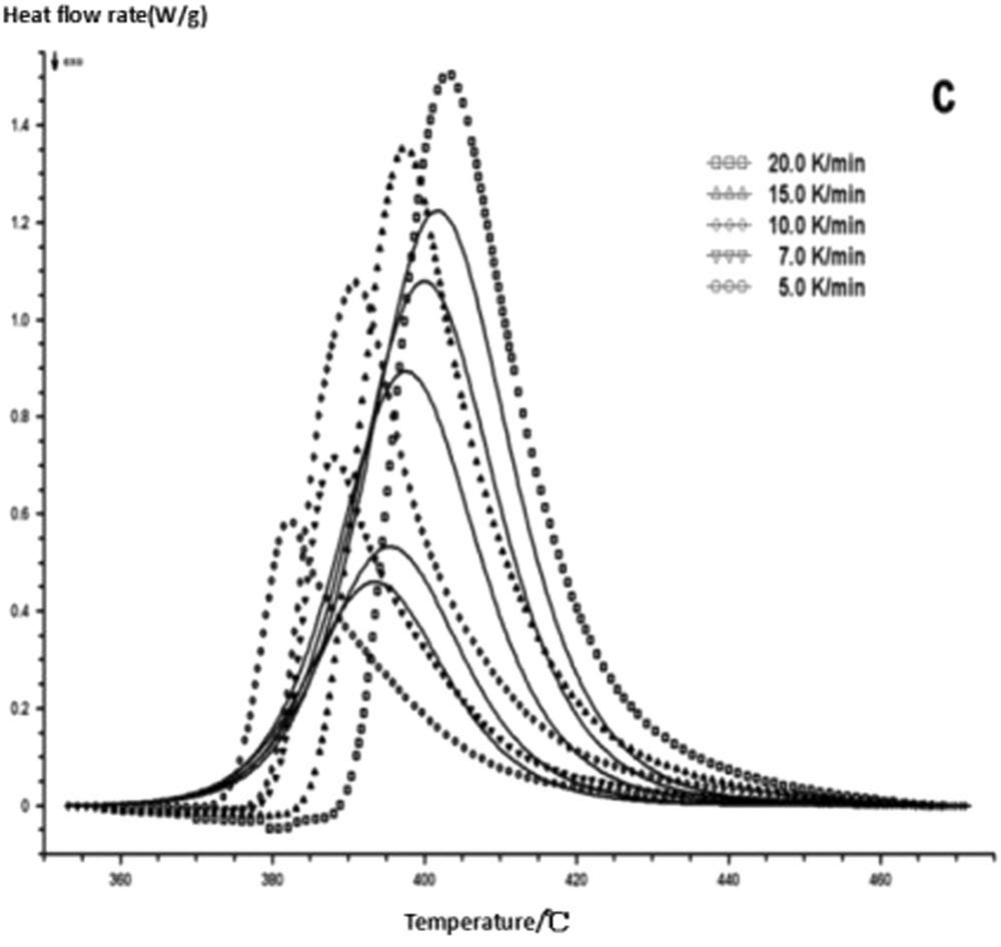
Reaction mechanism function fitting curve-c.

**Figure 21:**
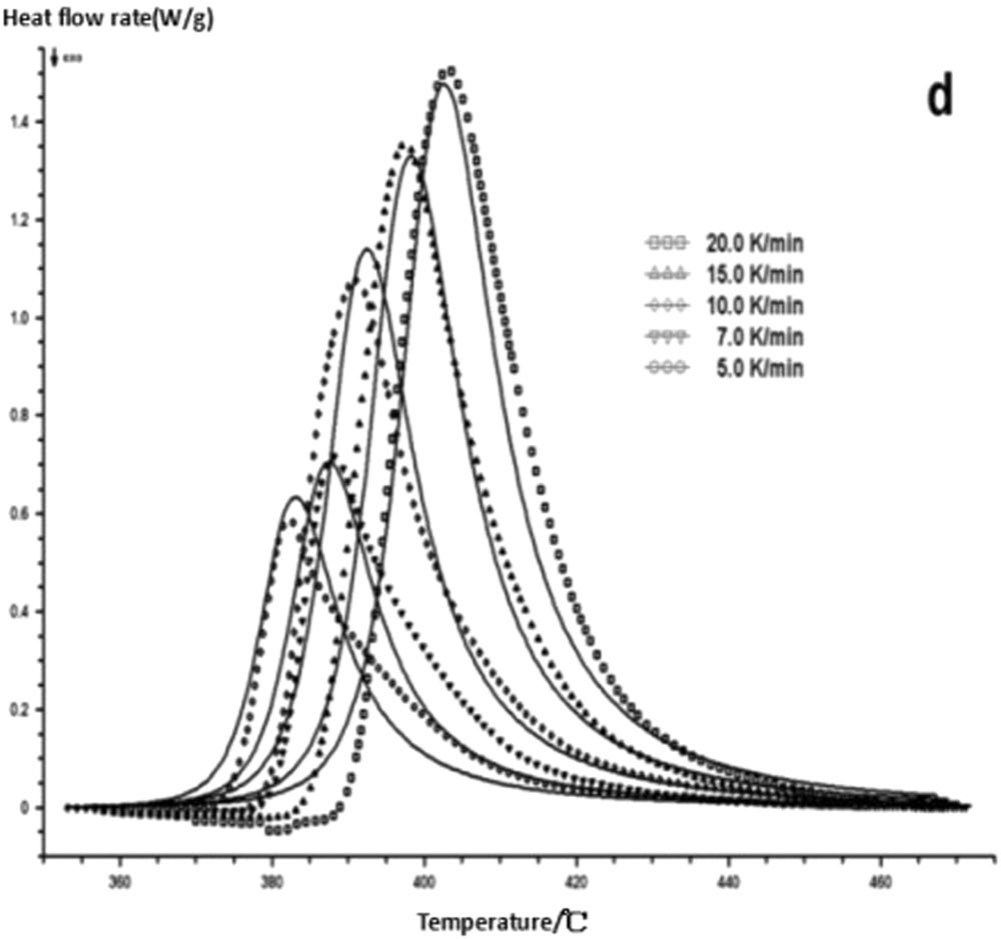
Reaction mechanism function fitting curve-d.

**Figure 22:**
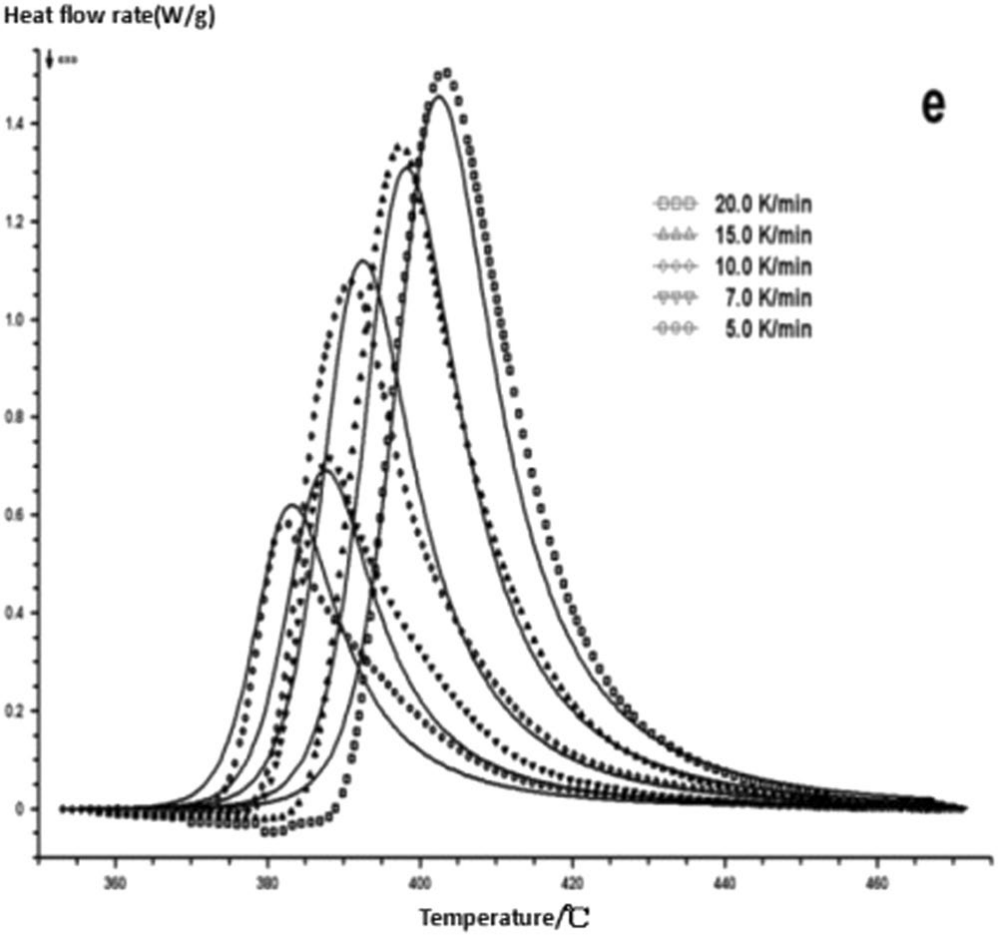
Reaction mechanism function fitting curve-e.

Comparing on fitting curves and test curves, the result is shown in Figs 18–22, the solid line is the fitting curve, and the dot matrix curve is the actual measurement data.

From the chart it can clearly seen that the curing system of DSC curve to simulate the PT kinetic mechanism functions coincide with test results of DSC. In the meantime, reaction mechanism corresponds to the self catalytic reaction shows that nano modified epoxy resin and six hexafluoroantimonate ammonium curing reaction proceeds in accordance with the autocatalytic reaction and it is consistent with the previous reaction mechanism.

## Conclusion

The curing curves of the two epoxy resin systems show that the peak of heating becomes steeper with the heating rate increasing, and the curing time is shorter. The curing characteristic temperature of the system moves to the high temperature.

After the addition of nanoparticles, the heat release was reduced and the time was shortened, indicating that the nanoparticles played a catalytic role in the reaction.

Three kinds of analytical methods were used to study the curing kinetics of the two curing systems, and the kinetic apparent activation energy E and the pre - exponential factor A were obtained.

The results show that the apparent activation energy and pre - exponential factors of the system are greatly reduced after the addition of the nanoparticles, which indicates that the nanoparticles can promote the curing reaction of the system and reduce the apparent activation energy of the system.

The kinetic model of nano-phase modified epoxy resin was fitted by multiple linear analysis method and different reaction mechanism. The results showed that the curing reaction mechanism was autocatalytic reaction.
